# A Versatile Multiple Target Detection System Based on DNA Nano-assembled Linear FRET Arrays

**DOI:** 10.1038/srep26879

**Published:** 2016-05-27

**Authors:** Yansheng Li, Hongwu Du, Wenqian Wang, Peixun Zhang, Liping Xu, Yongqiang Wen, Xueji Zhang

**Affiliations:** 1 School of Chemistry & Biological Engineering, University of Science and Technology Beijing, Beijing, 100083, China; 2 Peking University People’s Hospital, Beijing, 100044, China

## Abstract

DNA molecules have been utilized both as powerful synthetic building blocks to create nanoscale architectures and as inconstant programmable templates for assembly of biosensors. In this paper, a versatile, scalable and multiplex detection system is reported based on an extending fluorescent resonance energy transfer (FRET) cascades on a linear DNA assemblies. Seven combinations of three kinds of targets are successfully detected through the changes of fluorescence spectra because of the three-steps FRET or non-FRET continuity mechanisms. This nano-assembled FRET-based nanowire is extremely significant for the development of rapid, simple and sensitive detection system. The method used here could be extended to a general platform for multiplex detection through more-step FRET process.

High efficient detection of multiple biological/chemical molecules in complex conditions at one time is particularly important for early disease diagnosis, therapy, environmental monitoring, and food safety, etc. It has motivated intense interest in developing rapid, simple, and cost-effective nanoscale biosensing for proteins, nucleic acids, and small molecules^1^,^2^,^3^,^4^,^5^,^6^,^7^. However, much attention has currently been drawn toward the development of nanostructure platform for single target detection. Meanwhile, for most of the real scenario, the detection system would be quite complex, and contain numerous molecular species^8^,^9^. Therefore, to develop a biosensor in nanoscale that can yield signals in response to multiple components in one pot still remains a challenging task, which could reduce the amount of the sample and analytical operation and lead to a rapid, convenient analysis.

Fluorescent resonance energy transfer (FRET) is the most common photophysical process currently investigated, which provides information on the distance between a donor and an acceptor dye in the range of 10 to 100 Å. Optically addressed biosensors often use FRET for signal transduction by virtue of its high sensitivity^10^,^11^,^12^. Recently, sequential FRET has been reported by controlling the relative position of multiple fluorophores along a molecular scaffold with demonstrated strategies^13^,^14^,^15^,^16^. Inspired by such photonic wires, we expected to design some linear optical sensing devices at nanoscale which could realize multiplex detections simultaneously with high sensitivity and selectivity.

DNA nanotechnology has been seen as a fast evolving field in recent years due to the unique physicochemical properties, its specific ability to noncovalently self-assemble into complex yet predictable structures, and easy access to building-block oligomeric components via customizable, automated synthetic chemistry that allows for multiple site-specific chemical modifications^17^,^18^,^19^,^20^,^21^,^22^,^23^. DNA-based architectures and functional devices are currently playing an increasing role under investigation including molecular scale biosensors^24^,^25^,^26^,^27^,^28^,^29^,^30^,^31^. Such DNA-based biosensors usually consist of fluorophore quencher pairs and rely on FRET, in which distance-dependent fluorescence quenching is elaborately designed to be closely associated with DNA hybridization events. In the previous studies, we have used DNA as the template to precisely control the assembly of gold nanoparticles and fluorescence groups^32^,^33^,^34^,^35^,^36^. Given the capacity for precise control of fluorophore placement in DNA structures, FRET-based DNA photonic networks are especially relevant for multiplex detection applications.

In this communication, a versatile, scalable and multiplex detection system was reported based on an extending FRET cascades on a linear DNA photonic-wire. To prove this concept, up to four different probes (P1, P2, P3, P4) were coupled together to form the sequential FRET detection system. As shown in Fig. 1, four kinds of dye modified DNA probes with different sequences were designed. Four kinds of dyes (AMCA (D1), FAM (D2), Cy3 (D3) and ROX (D4)) with overlapping absorption and emission spectra (shown in Supporting Information Fig. S1) were modified on the probes and utilized as the photo-energy donor, acceptor or mediator, respectively^37^,^38^,^39^. Using such platform, seven combinations of three kinds of targets could be successfully detected through the changes of fluorescence spectra before and after the addition of targets. This FRET-based versatile detection system is extremely significant for the development of rapid, simple and sensitive detection platform. The method could be extended to a general platform for multiplex detection through more-step FRET by attaching more probes with overlapping absorption and emission spectra.

## Results and Discussion

The sequences of DNA probes are shown in Table S1, and their MALDI–TOF mass spectrometric analysis are shown in Fig. S2. The probes consist of two distinct functional domains. One domain is the probe sequence, which could sense and bind the target sequence, and the other domain is the connecting sequence, which is complementary to adjacent probes and could form stable duplex sequences. The stepwise formation of the DNA nanowire structures of the hybridization structure of four probes (P1, P2, P3, P4, P1-P2, P1-P2-P3, P1-P2-P3-P4) were verified by PAGE under non-denaturing conditions (Fig. S3). The single strand P1, P2, P3 and P4 alone showed a band of relatively high mobility. However, the assemblies of four building blocks showed a single band of steadily decreasing mobility, indicating that a well-defined hybridization structure was formed. Through the sequential arrangement of dye in an appropriate space and orientation, a highly efficient multistep FRET-based detection system could be constructed through the noncovalent DNA recognition in aqueous media.

**Figure 2:**
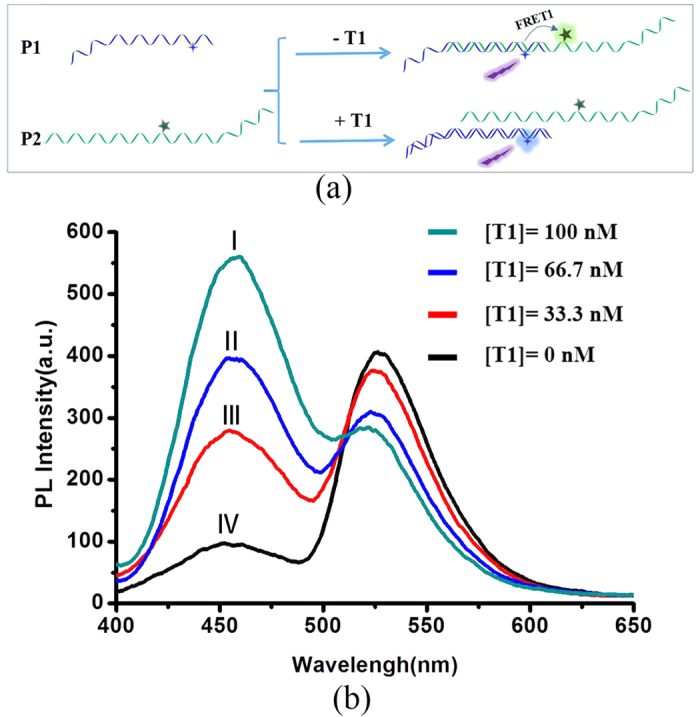
(**a**) Schematic representation of single target DNA (T1) detection procedure using a two-DNA sequence assembled system. (**b**) Representative fluorescence spectra of the system consist of P1 (100 nM) and P2 (100 nM) in the presence of various concentrations of target T1. Excitation wavelength: 364 nm.

As the simplest example, when the system consists of only two oligo-DNA sequences, the system could be used to detect one kind of target. As shown in Fig. 2a, in the absence of target 1 (T1), probe P1 and P2 would be partly hybridized, which would lead to the quenching of fluorescence from donor chromophores (D1) and the increasing of fluorescence from acceptor chromophores (D2) due to the FRET effect (Fig. 2b, curve IV). However, when T1, which is fully complementary to P1, was added, it will bind strongly to P1, replace P2 in the P1-P2 conjugates, and effective FRET could be decreased. The corresponding fluorescence spectra changes are shown in Fig. 2b (curve I, II, III). The fluorescence of D1 was intensified along with the increase of the target concentration, and the fluorescence of D2 was decreased at the same time. The same experiment was also performed for the combination of P2-P3 and P3-P4 (Supporting Information, Fig. S4). These results indicated that single-step FRET could be used to detect single DNA from the changes of fluorescence that were distinguishable from the background.

**Figure 3:**
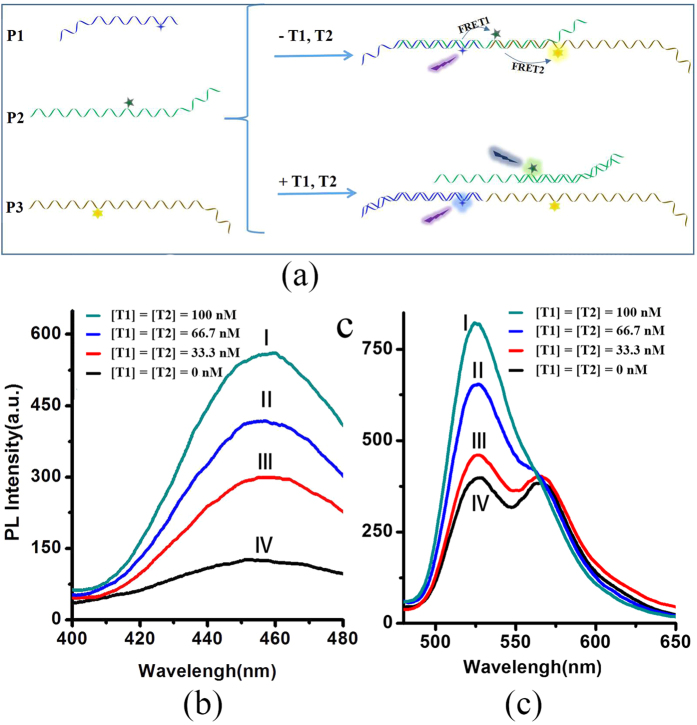
(**a**) Schematic representation of simultaneous detection procedures of two specific target DNA (T1 and T2) using a three-DNA sequence assembled system; (**b**,**c**) fluorescence spectra of two-step FRET detection system (P1-P2-P3) in the presence of various concentrations of targets (T1 and T2). Excitation wavelengths: (**b**) 364 nm; (**c**) 457 nm.

Two-step FRET detection system were further investigated. A schematic illustration of the experimental procedure is shown in Fig. 3. In the absence of target, P1, P2 and P3 could generate an assembled structure by partly complementary pairing between each other. And at this situation, effective FRET1 and FRET2 occurred between P1/P2 and P2/P3 donor-acceptor pairs. When target strand T1 and T2, which possess a sequence that is fully complementary to the P1 and P2 respectively, are added, the system would form the combinations of T1/P1 and T2/P2, and the chromophores moving far away from each other (Fig. 3a), and no effective FRETs occurred. Different excitation wavelengths (364nm, 457nm) were used to detect the effective changes of FRET1 and FRET2 as the targets were added. Different fluorescence changes were exhibited when only one or both targets were simultaneously added. As shown in Fig. 3b,c, when T1 and T2 were added simultaneously, the fluorescence of D1 greatly increased under 364 nm excitation wavelengths (Fig. 3b), and at the same time the increase of fluorescence from D2 and the decline of fluorescence from D3 were observed at an excitation wavelength of 457 nm (Figs 3 and S5). Furthermore, the changes of fluorescence intensity increase with the increase of the concentration of T1 and T2. At the cases of only one target present, the increase of fluorescence from D1 were observed on addition of only T1 under 364 nm excitation wavelengths, and at the same time, no obvious changes of fluorescence from D2 and D3 were found at an excitation wavelength of 457 nm. Comparatively, the increase of fluorescence from D2 and the decline of D3 were observed when only T2 was added, and no changes of fluorescence from D1, as shown in supporting information (Fig. S6). The fact that obvious fluorescence changes from P1-P2-P3 conjugates with different targets was observed at an excitation wavelength of 364 nm and 457 nm indicates that two targets could be detected by the two-step FRET detection platform.

**Figure 4:**
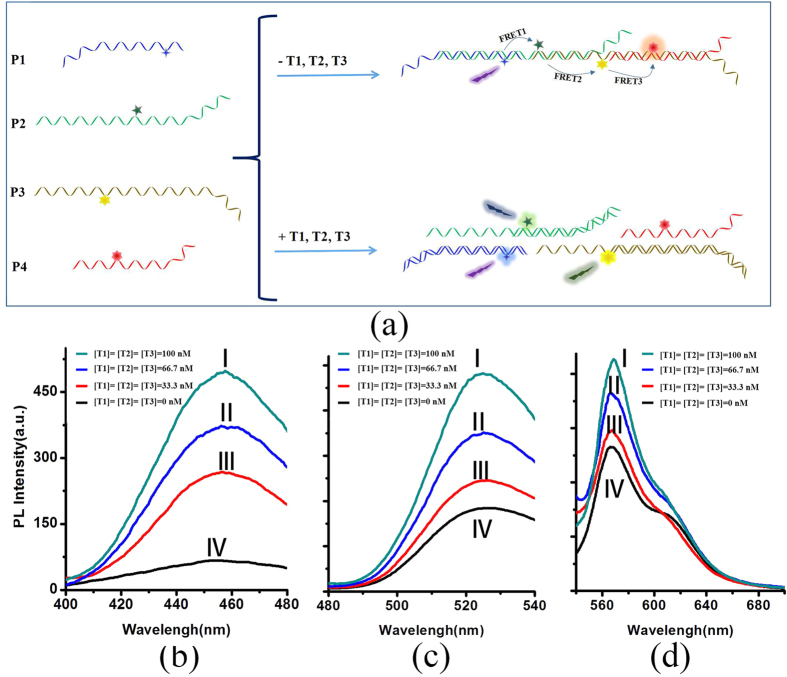
(**a**) Schematic representation of simultaneous detection procedures of three specific targets by a three-DNA sequence assembled system; (**b–d**) fluorescence spectra for three-step FRET detection platform (P1-P2-P3-P4) in the presence of various concentrations of targets. Excitation wavelengths: (**b**) 364 nm; (**c**) 457 nm; (**d**) 520 nm.

Three-step FRET based detection platform was further investigated through a more complex P1-P2-P3-P4 system. Firstly, the mixed solution of P1, P2, P3 and P4 (in 1:1:1:1 ratio) was prepared. As shown in Fig. 4a, in the absence of target, P1, P2, P3 and P4 could self-assemble into an assembled structure from these flexible components by partly complementary pairing between each other. At this situation, effective FRET1, FRET2 and FRET3 would happen between P1/P2, P2/P3 and P3/P4 donor-acceptor pairs (Fig. 4a). When the solution system was titrated with different target or target combination, it would display obvious different fluorescence spectra. As shown in Fig. 4a, when the target mixture (T1, T2, T3) were added, no FRET steps occurred. Correspondingly, the increase of fluorescence from D1 (Fig. 4b), D2 (Fig. 4c), D3 (Fig. 4d), and the decline of fluorescence from D4 (Figs 4d and S7) were observed. Furthermore, through this three-step FRET based detection platform, seven kinds of target combinations could be identified, which were one-target (T1, T2, T3), combinations of two targets (T1-T2, T2-T3, T1-T3) and combinations of three targets (T1-T2-T3), respectively. The typical results of single target and two target combinations (here, single target T1 and two targets T1\T3 combinations was selected as the examples) were shown in supporting information (Fig. S8). From the fluorescence spectra, the increase of fluorescence from D1 were observed on addition of only T1 under 364 nm excitation wavelengths, and no changes of fluorescence from D2, D3, D4 were found at an excitation wavelength of 457 nm and 520 nm, as shown in supporting Fig. S8b, c, d). As shown in supporting Fig. S8f–h, the fluorescence of D1 and D3 become stronger, and D2 display no change when T1 and T3 were added simultaneously. Based on the changes of fluorescence spectra, the detection results of seven kinds of combinations of three targets were summarized in Table 1.

**Figure 5:**
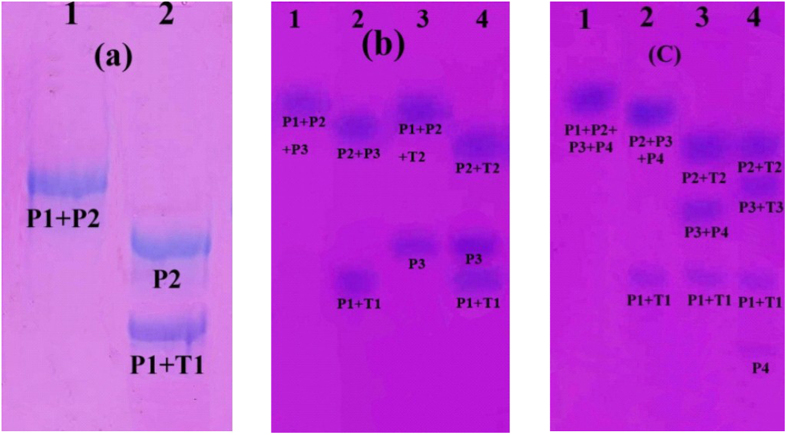
Native polyacrylamide gel electrophoresis (PAGE) (7%, 1 × TAEMg) analysis of the detection systems. (**a**) The representative two probe-composed system (P1-P2) for one target (T1) detection. Lane 1: gel confirmation of the quantitative hybridization of probe P1 and P2, Lane 2: PAGE analysis of the P1-P2 system in the presence of target stand T1; (**b**) The representative three probe-composed system (P1-P2-P3) for two target (T1 or/and T2) detection. Lane 1: gel confirmation of the quantitative hybridization of probe P1, P2 and P3, Lane 2 (or Lane 3): PAGE analysis of the P1-P2-P3 system in the presence of target stand T1 (or T2), Lane 4: PAGE analysis of the P1-P2-P3 system in the presence of target stands T1 and T2; (**c**) The representative four probe-composed system (P1-P2-P3-P4) for three target (T1 or/and T2 or/and T3) detection. Lane 1: gel confirmation of the quantitative hybridization of probe P1, P2, P3 and P4, Lane 2: PAGE analysis of the P1-P2-P3-P4 system in the presence of target stand T1, Lane 3: PAGE analysis of the P1-P2-P3-P4 system in the presence of target stand T1 and T2, Lane 4: PAGE analysis of the P1-P2-P3-P4 system in the presence of target stand T1, T2 and T3.

The above fluorescence analysis results could be further verified by polyacrylamide gel electrophoresis (PAGE) under non-denaturing conditions. As the examples, Fig. 5 shows the PAGE analysis results of the three representative situations, i.e. two probe-composed system for one target (a), three probe-composed system for two target detection (b), and four probe-composed system for three target detection (c), respectively. As shown in Fig. 5, the different assembles in the analysis system showed different bands due to their different mobility. The PAGE analysis result was consistent with the corresponding fluorescence analysis and diagrammatic sketch.

In summary, a versatile DNA nanowire based on extending FRET linearly arrays was successfully assembled with four dye-DNA probes. The fluorescence spectroscopy produced significant changes before and after the addition of different target combinations into the detection system because of the DNA nanowire was broke by target. Furthermore, specific aptamer molecules could bind to various specific target species, such as small organics, peptides, proteins, and metal ions with high affinity and specificity, this method could be extended to a long nanowire for multiplex detection through such multiple step FRET system, which could distinguish more targets and their combinations theoretically.

## Experimental Section

Standard automated oligonucleotide solid-phase synthesis was performed on a BioAutomation MerMade 4 DNA synthesizer. UV-Vis spectra were measured on a Shimadzu U-1800 spectrophotometer. High-performance liquid chromatography (HPLC) was performed using an Elite P230PII series HPLC. Gel electrophoresis experiments were carried out on an acrylamide 20 × 20 cm vertical DYCZ24 electrophoresis unit. All fluorescence spectra were recorded on a Hitachi F-4500 FL Spectrophotometer in PBS buffer.

The oligonucleotides were constructed on CPG supports using conventional phosphoramidite chemistry. For the sequences modified by fluorescence group in the interior or at the end of the DNA sequence, the coupling and deprotection times were extended to eight and two minutes, respectively. Products were cleaved from the support by treatment with concentrated NH_4_OH for 16 h at 55 °C. The NH_4_OH solution was decanted and dried down to yield the crude DNA mixture. The crude mixture obtained was purified by preparative reverse-phase HPLC with 0.03 M triethylammonium acetate (TEAA), pH 7 and a 1%/min gradient of 95% CH_3_CN/5% 0.03 M TEAA at a flow rate of 1 mL/min. Quantification was estimated based on UV-Vis absorbance at 260 nm.

The representative extending FRET-based DNA detection system (200 mM NaCl, 25 mM Tris acetate, pH 8.2) containing various probe combinations (with 100 nM P1 and/or P2 and/or P3 and/or P4) was annealed in the presence or absence of a certain concentration DNA targets (T1, T2, T3 and their combinations) from 90 °C to 20 °C, then stored at this temperature over 2 h. All fluorescence measurements were initially carried out at 20 °C in above buffer system. Annealed structures were further verified by polyacrylamide gel electrophoresis (PAGE) under non-denaturing conditions. All of the final assemblies were characterized using 7% native polyacrylamide gel (run at constant current of 10 mA, 4 °C and visualized using StainsAll^®^).

## Additional Information

**How to cite this article**: Li, Y. *et al.* A Versatile Multiple Target Detection System Based on DNA Nano-assembled Linear FRET Arrays. *Sci. Rep.* **6**, 26879; doi: 10.1038/srep26879 (2016).

## Supplementary Material

Supplementary Information

## Figures and Tables

**Figure 1:**
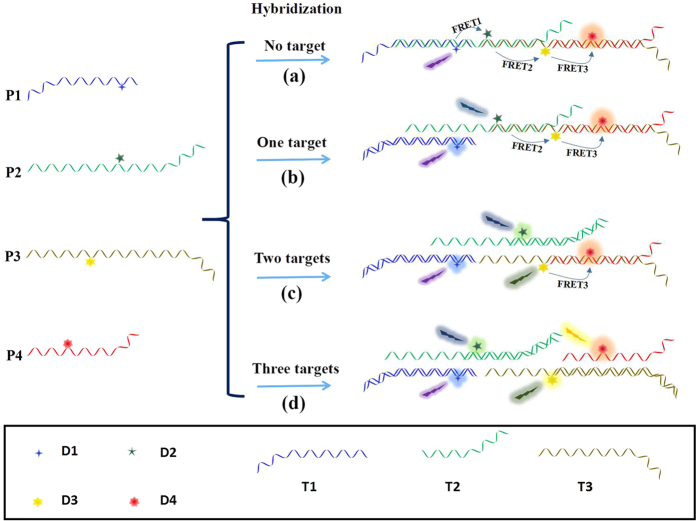
Scheme of multiple detection platforms based on DNA assembled linear extending FRET. P1, P2, P3, P4 represent different probes, which consist of designed DNA sequences, and D1, D2, D3, D4 represent different dyes attached to the probes, respectively. (**a**) In the absence of target (no target), P1, P2, P3 and P4 could self-assemble into a linear structure, leading to successive fluorescence resonance energy transfer (FRET) between the donors and acceptors, i.e. FRET1, FRET2 and FRET3; (**b**) When target sequence T1 was added, the effective FRET1 would be weakened; (**c**) When target sequences T1 and T2 were simultaneously added, the effective FRET1 and FRET2 would be weakened; (**d**) When target sequences T1, T2 and T3 were simultaneously added, the effective FRET1, FRET2 and FRET3 would be weakened.

**Table 1:** Summary of the fluorescence changes in the three-step FRET detection system with the addition of different target combination.

	D1	D2	D3	D4
T1	+			
T2		+		
T3			+	−
T1+T2	+	+		
T1+T3	+		+	−
T2+T3		+	+	−
T1+T2+T3	+	+	+	−

“**−**” represent fluorescence decline and “**+**” represent fluorescence increase.

## References

[b1] Mao S. *et al.* Direct growth of vertically-oriented graphene for field-effect transistor biosensor. Scientific reports 3, 1696, (2013). 23603871 10.1038/srep01696 PMC3631944

[b2] Gao W. & Wang J. Synthetic micro/nanomotors in drug delivery. Nanoscale 6, 10486, (2014). 25096021 10.1039/c4nr03124e

[b3] Wang F. & Liu J. Evaporation induced wrinkling of graphene oxide at the nanoparticle interface. Nanoscale 7, 919–923, (2015). 25475682 10.1039/c4nr05832a

[b4] Cibulskis K. *et al.* Sensitive detection of somatic point mutations in impure and heterogeneous cancer samples. Nature Biotechnology 31, 213–219, (2013). 10.1038/nbt.2514 PMC3833702 23396013

[b5] Elghanian R. Selective Colorimetric Detection of Polynucleotides Based on the Distance-Dependent Optical Properties of Gold Nanoparticles. Science 277, 1078–1081, (1997). 9262471 10.1126/science.277.5329.1078

[b6] Nam J. M. Nanoparticle-Based Bio-Bar Codes for the Ultrasensitive Detection of Proteins. Science 301, 1884–1886, (2003). 14512622 10.1126/science.1088755

[b7] Park S. J., Taton T. A. & Mirkin C. A. Array-based electrical detection of DNA with nanoparticle probes. Science 295, 1503–1506, (2002). 11859188 10.1126/science.1067003

[b8] Unnikrishnan B., Palanisamy S. & Chen S.-M. A simple electrochemical approach to fabricate a glucose biosensor based on graphene–glucose oxidase biocomposite. Biosensors and Bioelectronics 39, 70–75, (2013). 22795531 10.1016/j.bios.2012.06.045

[b9] Haes A. J. & Van Duyne R. P. A Nanoscale Optical Biosensor: Sensitivity and Selectivity of an Approach Based on the Localized Surface Plasmon Resonance Spectroscopy of Triangular Silver Nanoparticles. Journal of the American Chemical Society 124, 10596–10604, (2002). 12197762 10.1021/ja020393x

[b10] Clegg R. M. Fluorescence resonance energy transfer. Current Opinion in Biotechnology 6, 103–110, (1995). 7534502 10.1016/0958-1669(95)80016-6

[b11] Piston D. W. & Kremers G.-J. Fluorescent protein FRET: the good, the bad and the ugly. Trends in Biochemical Sciences 32, 407–414, (2007). 17764955 10.1016/j.tibs.2007.08.003

[b12] Wei L. *et al.* FRET ratiometric probes reveal the chiral-sensitive cysteine-dependent H2S production and regulation in living cells. Scientific Reports 4, (10.1038/srep04521)(2014).

[b13] Abraham B. G., Santala V., Tkachenko N. V. & Karp M. Fluorescent protein-based FRET sensor for intracellular monitoring of redox status in bacteria at single cell level. Analytical and Bioanalytical Chemistry 406, 7195–7204, (2014). 25224640 10.1007/s00216-014-8165-1

[b14] Song E. *et al.* A graphene oxide-based FRET sensor for rapid and sensitive detection of matrix metalloproteinase 2 in human serum sample. Biosensors and Bioelectronics 47, 445–450, (2013). 23623988 10.1016/j.bios.2013.03.030

[b15] Spillmann C. M. *et al.* Extending FRET cascades on linear DNA photonic wires. Chemical Communications 50, 7246, (2014). 24752334 10.1039/c4cc01072h

[b16] Buckhout-White S. *et al.* Assembling programmable FRET-based photonic networks using designer DNA scaffolds. Nature Communications 5, 5615, (2014). 10.1038/ncomms6615 PMC4275599 25504073

[b17] Dong Y., Yang Z. & Liu D. DNA Nanotechnology Based on i-Motif Structures. Accounts of Chemical Research 47, 1853–1860, (2014). 24845472 10.1021/ar500073a

[b18] He S. *et al.* A Graphene Nanoprobe for Rapid, Sensitive, and Multicolor Fluorescent DNA Analysis. Advanced Functional Materials 20, 453–459, (2010).

[b19] Banerjee A. *et al.* Controlled Release of Encapsulated Cargo from a DNA Icosahedron using a Chemical Trigger. Angewandte Chemie International Edition 52, 6854–6857, (2013). 10.1002/anie.201302759 23716499

[b20] Olejko L., Cywinski P. J. & Bald I. Ion-selective formation of a guanine quadruplex on DNA origami structures. Angew Chem Int Ed Engl 54, 673–677, (2015). 25413669 10.1002/anie.201409278

[b21] Amodio A. *et al.* Rational Design of pH-Controlled DNA Strand Displacement. Journal of the American Chemical Society 136, 16469–16472, (2014). 25369216 10.1021/ja508213d

[b22] Sternberg S. H., Redding S., Jinek M., Greene E. C. & Doudna J. A. DNA interrogation by the CRISPR RNA-guided endonuclease Cas9. Nature 507, 62–67, (2014). 24476820 10.1038/nature13011 PMC4106473

[b23] Liu J. & Lu Y. Preparation of aptamer-linked gold nanoparticle purple aggregates for colorimetric sensing of analytes. Nature Protocols 1, 246–252, (2006). 17406240 10.1038/nprot.2006.38

[b24] Sakuma T. *et al.* Repeating pattern of non-RVD variations in DNA-binding modules enhances TALEN activity. Scientific reports 3, 3379, (2013). 24287550 10.1038/srep03379 PMC3843162

[b25] Zhou W., Jimmy Huang P.-J., Ding J. & Liu J. Aptamer-based biosensors for biomedical diagnostics. The Analyst 139, 2627, (2014). 24733714 10.1039/c4an00132j

[b26] Wu J. *et al.* Cyclic GMP-AMP is an endogenous second messenger in innate immune signaling by cytosolic DNA. Science 339, 826–830, (2013). 23258412 10.1126/science.1229963 PMC3855410

[b27] Lu C. H., Willner B. & Willner I. DNA nanotechnology: from sensing and DNA machines to drug-delivery systems. ACS nano 7, 8320–8332, (2013). 24070191 10.1021/nn404613v

[b28] Niazov-Elkan A., Golub E., Sharon E., Balogh D. & Willner I. DNA Sensors and Aptasensors Based on the Hemin/G-quadruplex-Controlled Aggregation of Au NPs in the Presence of L-Cysteine. Small 10, 2883–2891, (2014). 24700798 10.1002/smll.201400002

[b29] Liu J., Cao Z. & Lu Y. Functional nucleic acid sensors. Chemical reviews 109, 1948–1998, (2009). 19301873 10.1021/cr030183i PMC2681788

[b30] Song S. *et al.* Gold-Nanoparticle-Based Multicolor Nanobeacons for Sequence-Specific DNA Analysis. Angewandte Chemie International Edition 48, 8670–8674, (2009). 10.1002/anie.200901887 19731289

[b31] Monroe K. M. *et al.* IFI16 DNA Sensor Is Required for Death of Lymphoid CD4 T Cells Abortively Infected with HIV. Science 343, 428–432, (2014). 24356113 10.1126/science.1243640 PMC3976200

[b32] Wen Y. *et al.* A flexible DNA modification approach towards construction of gold nanoparticle assemblies. Chemical Communications 48, 3963–3965, (2012). 22422147 10.1039/c2cc30846k

[b33] Chen L. *et al.* A pH-driven DNA nanoswitch for responsive controlled release. Chemical Communications 47, 2850–2852, (2011). 21253628 10.1039/c0cc04765a

[b34] Chen L. *et al.* Programmable DNA switch for bioresponsive controlled release. Journal of Materials Chemistry 21, 13811, (2011).

[b35] Wen Y., McLaughlin C. K., Lo P. K., Yang H. & Sleiman H. F. Stable gold nanoparticle conjugation to internal DNA positions: facile generation of discrete gold nanoparticle-DNA assemblies. Bioconjugate chemistry 21, 1413–1416, (2010). 20666441 10.1021/bc100160k

[b36] Wen Y. *et al.* Controllable and reproducible construction of a SERS substrate and its sensing applications. Nanoscale 5, 523–526, (2013). 23223828 10.1039/c2nr33350c

[b37] Kim S. *et al.* Deoxyribozyme-loaded nano-graphene oxide for simultaneous sensing and silencing of the hepatitis C virus gene in liver cells. Chemical Communications 49, 8241, (2013). 23926597 10.1039/c3cc43368d

[b38] Nagamine K., Watanabe K., Ohtsuka K., Hase T. & Notomi T. Loop-mediated isothermal amplification reaction using a nondenatured template. Clin Chem 47, 1742–1743, (2001). 11514425

[b39] Kim S. *et al.* Deoxyribozyme-loaded nano-graphene oxide for simultaneous sensing and silencing of the hepatitis C virus gene in liver cells. Chemical Communications 49, 8241–8243, (2013). 23926597 10.1039/c3cc43368d

