# Corrigendum: A Versatile Multiple Target Detection System Based on DNA Nano-assembled Linear FRET Arrays

**DOI:** 10.1038/srep28411

**Published:** 2016-07-07

**Authors:** Yansheng Li, Hongwu Du, Wenqian Wang, Peixun Zhang, Liping Xu, Yongqiang Wen, Xueji Zhang

Scientific Reports 6: Article number: 2687910.1038/srep26879; published online: 05272016; updated: 07072016

The Acknowledgements section in this Article is incomplete.

“This work is continuously supported by the National Natural Science Foundation of China (Grant nos 21171019, 51373023), and the Fundamental Research Funds for the Central Universities and NCET-11-0584.”

should read:

“This work is continuously supported by the National Natural Science Foundation of China (Grant nos 21171019, 51373023, 31271284, 31571235), and the Fundamental Research Funds for the Central Universities and NCET-11-0584.”

