# Supplementary material for: A Versatile Multiple Target Detection System Based on DNA Nano-assembled Linear FRET Arrays
Source: Sci Rep. 2016 May 27;6:26879. doi: 10.1038/srep26879 (PMC4882533; doi:10.1038/srep26879)
Supplement: Supplementary Information [file srep26879-s1.doc]

Supporting Information

A Versatile Multiple Target Detection System Based on DNA Nano-assembled Linear FRET Arrays

Yansheng Li^1^, Hongwu Du^1^, Wenqian Wang^1^, Peixun Zhang^2,*^, Liping Xu^1^, Yongqiang Wen^1^,*, Xueji Zhang^1^

^1^ School of Chemistry & Biological Engineering, University of Science and Technology Beijing, Beijing, 100083, China.

^2^ Peking University People’s Hospital, Beijing, 100044, China.

*****Email: Prof. Y. Q. Wen, wyq_wen@ustb.edu.cn and

Prof. P. X. Zhang, zhangpeixun@126.com

*Materials*

Boric acid, acetic acid, ethylenediamine-tetraacetic acid, NaCl, MgCl_2_.6H_2_O, StainsAll®, formamide, tris(hydroxymethyl)-aminomethane (Tris), and ethidium bromide, bis(*p-*sulfo natophenyl) phenylphosphine dihydrate dipotassium salt were purchased from Aldrich. 5-ethylthiotetrazole, controlled pore glass (CPG) and reagents for automated DNA syntheses were purchased from Beijing DNA Chem Biotechnology. All buffers were prepared with ultra-pure MilliQ water (resistance > 18 MΩ cm^-1^).


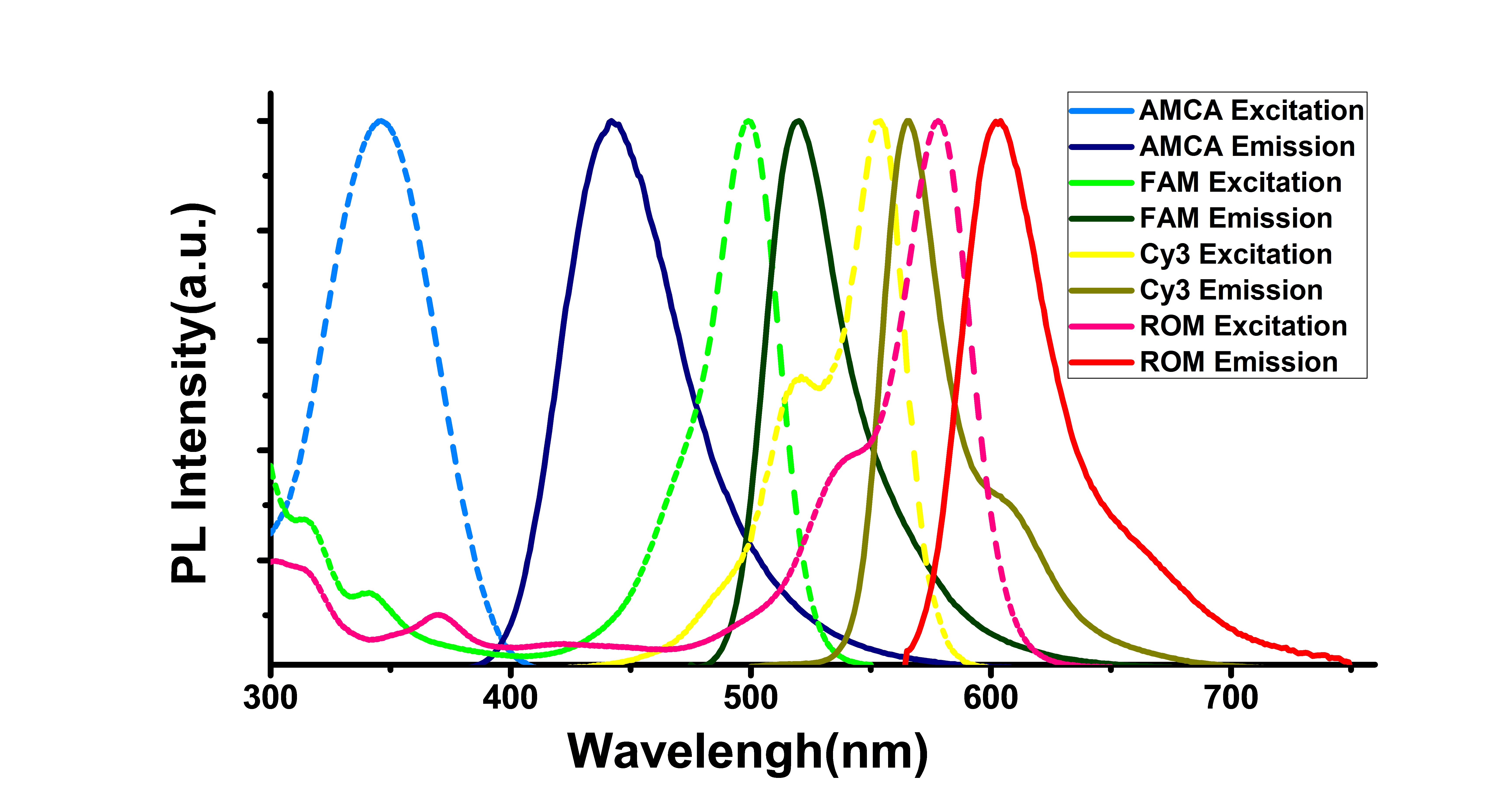


**Fig.S1** Normalized absorption and emission spectra of AMCA, FAM, Cy3 and ROM.

Table S1. DNA sequences of different probes and targets, dye placement and modifications

| Abbreviation | Structure(sequenced) | Modification |
| --- | --- | --- |
| P1 | 5'-AGGAACGTGTGGAAGG-3' | 5’ AMCA(D1) |
| P2 | 5’-CACACGTTCCTAAT*CATGTTTGTTGTTGGCCCCCCTTCTTTCTTA-3’ | Internal FAM(D2) |
| P3 | 5'-TGGAAGGAGGCGTTATGAGGGGGTCCAT*CAACAAACATGA-3' | Internal Cy3(D3) |
| P4 | 5'-GACCCCCTCAT-3' | 5’ ROX(D4) |
| T1 | 5'- CCTTCCACACCTTCC T -3' |  |
| T2 | 5'- TAAGAA AGA AGGGGGGCCAAC AA CAAACA TG -3' |  |
| T3 | 5'- ATGGAC CCC CTC ATA ACGCCTCCTTCC A -3' |  |
|  | * indicates modifier placement |  |


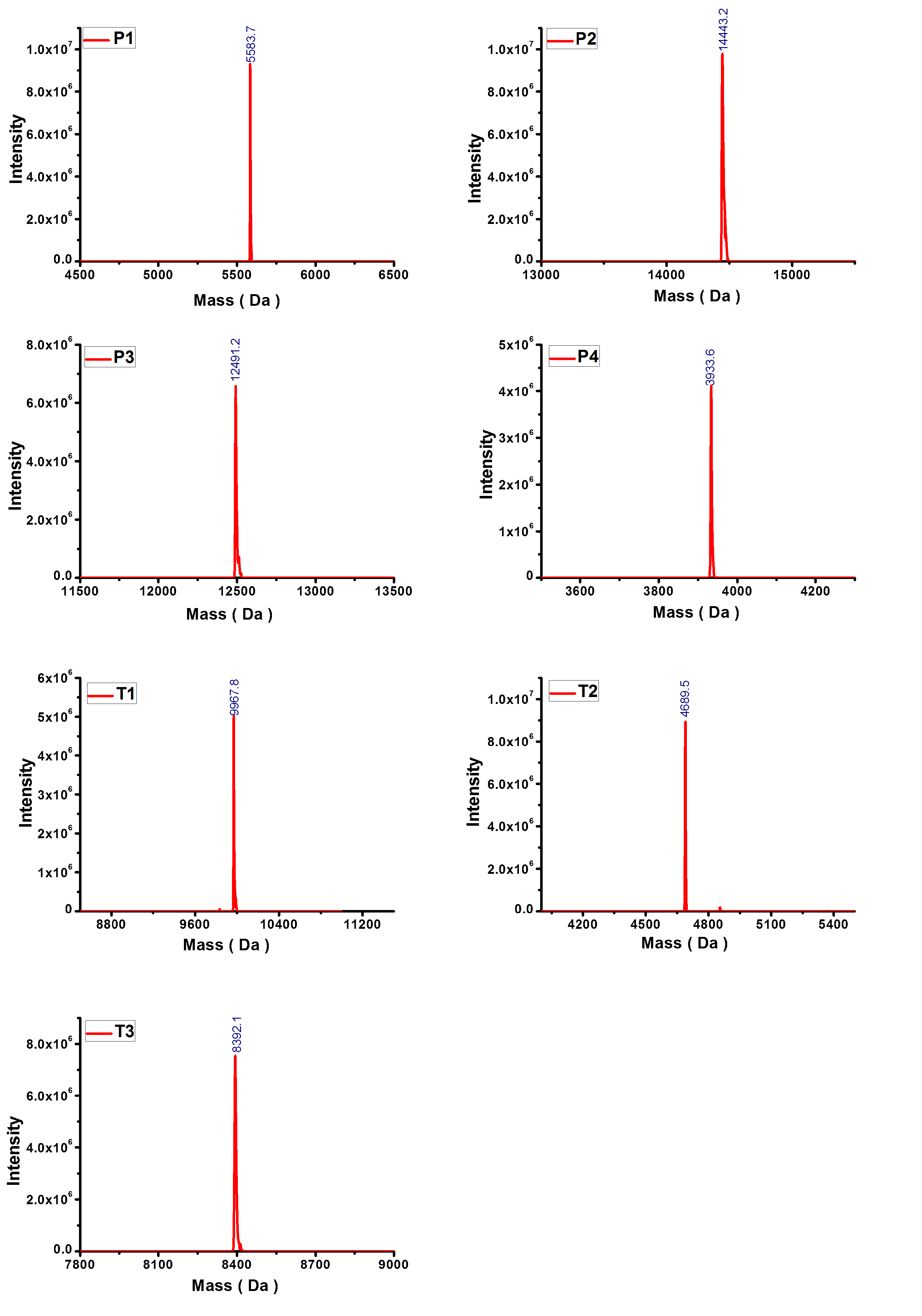


**Fig. S2** MALDI–TOF mass spectrometric analysis of the synthesized DNA strands P1, P2, P3, P4, T1, T2 and T3.


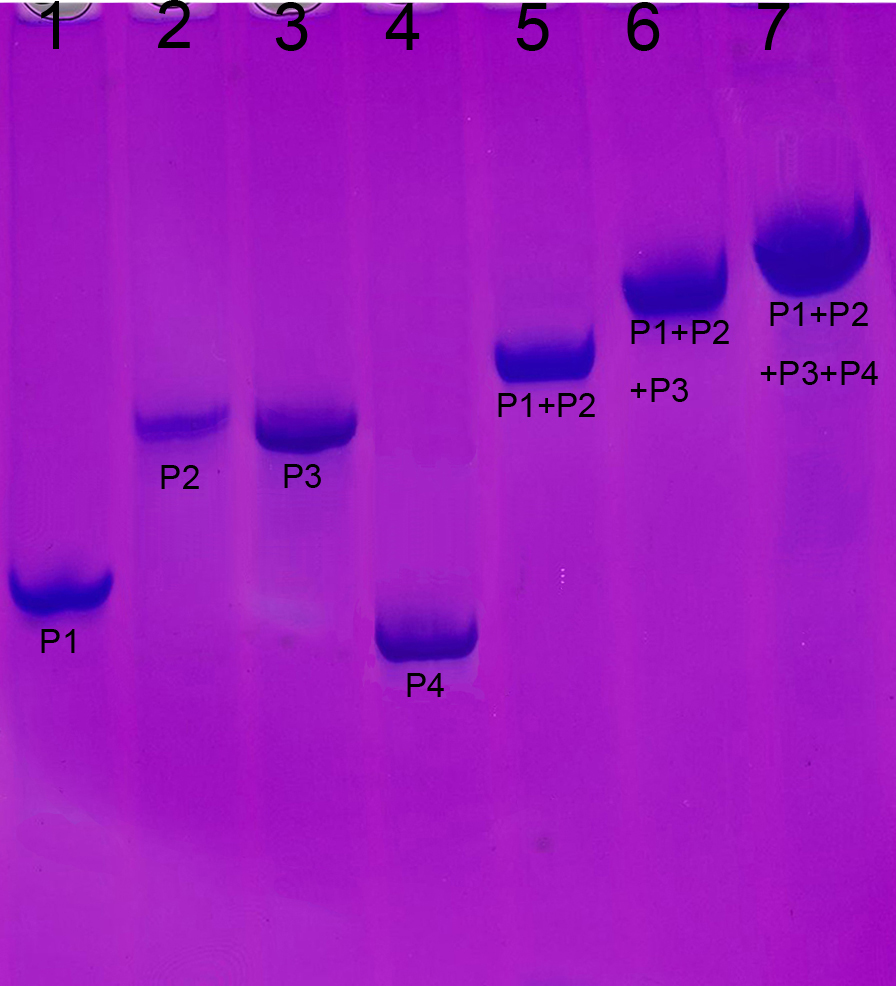


**Fig. S3** Native polyacrylamide gel (7%, 1x TAEMg) analysis of the assembly products. Lane 1, lane 2, lane 3, lane 4 are P1, P2, P3, P4, respectively. And lane 5, lane 6, lane 7 are the hybridization structure of P1-P2, P1-P2-P3 and P1-P2-P3-P4, which were assembled in Tris solution, and then annealed to yield the assembled nanostructure.


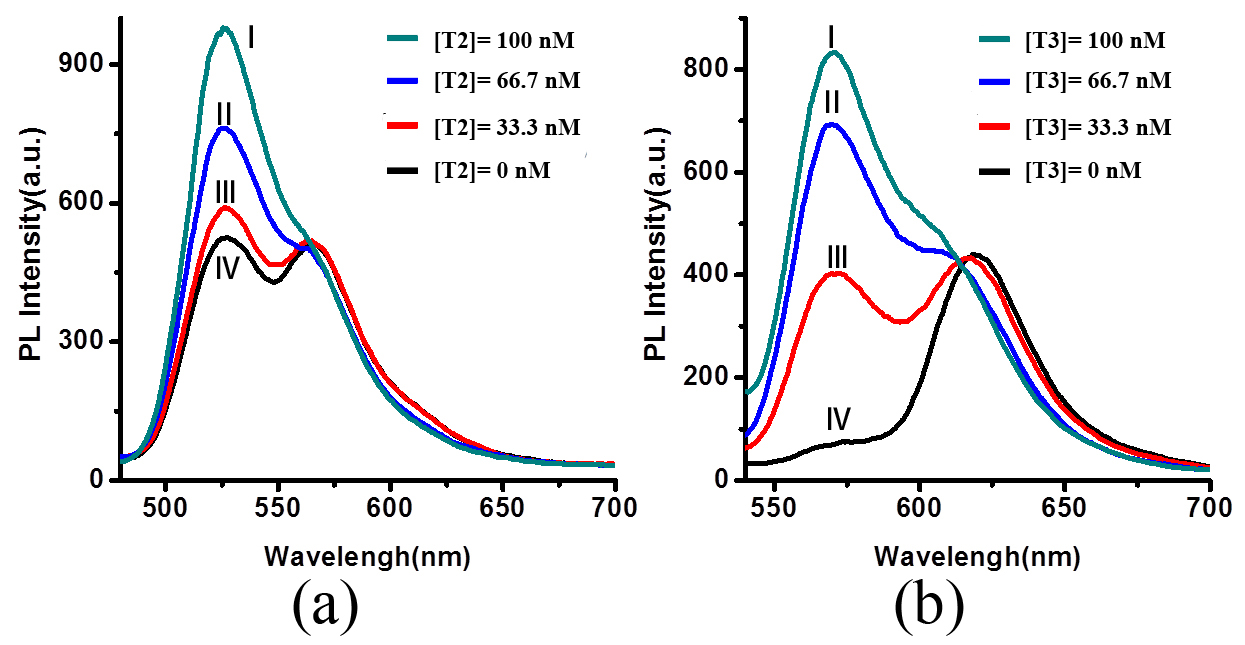


**Fig. S4** (a) Representative fluorescence spectra of the system consist of P2 (100 nM) and P3 (100 nM) in the presence of various concentrations of target T2. Excitation wavelength: 457 nm. (b) Representative fluorescence spectra of the system consist of P3 (100 nM) and P4 (100 nM) in the presence of various concentrations of target T3. Excitation wavelength: 520 nm.


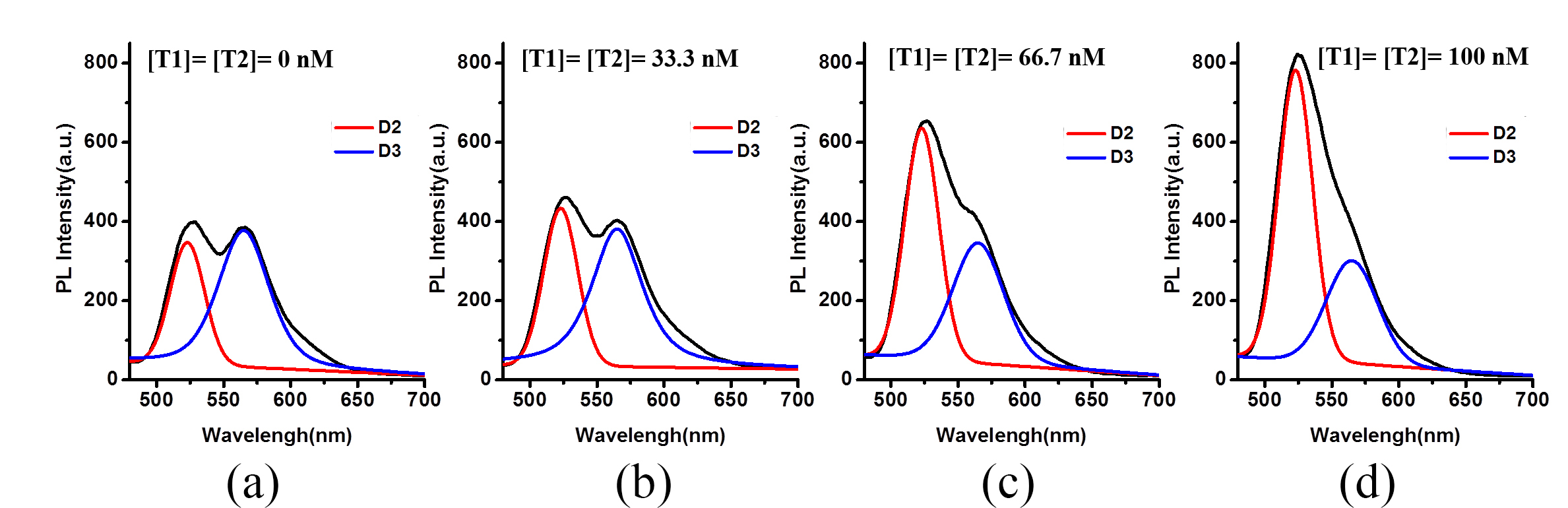


**Fig. S5** Fluorescence spectra of a three-DNA sequence assembled system (P1-P2-P3) for the [simultaneous](http://www.iciba.com/simultaneous) detection procedures of various concentrations of targets (T1 and T2). The experimental spectra (black curves) are fitted with two components by a weighted summation of the constituent D2 and D3 emission spectra. The resulting weighted contributions of the individual dyes D2 (red curve, at 523 nm) and D3 (blue curve, at 568 nm) are shown. Excitation wavelengths: 457 nm.


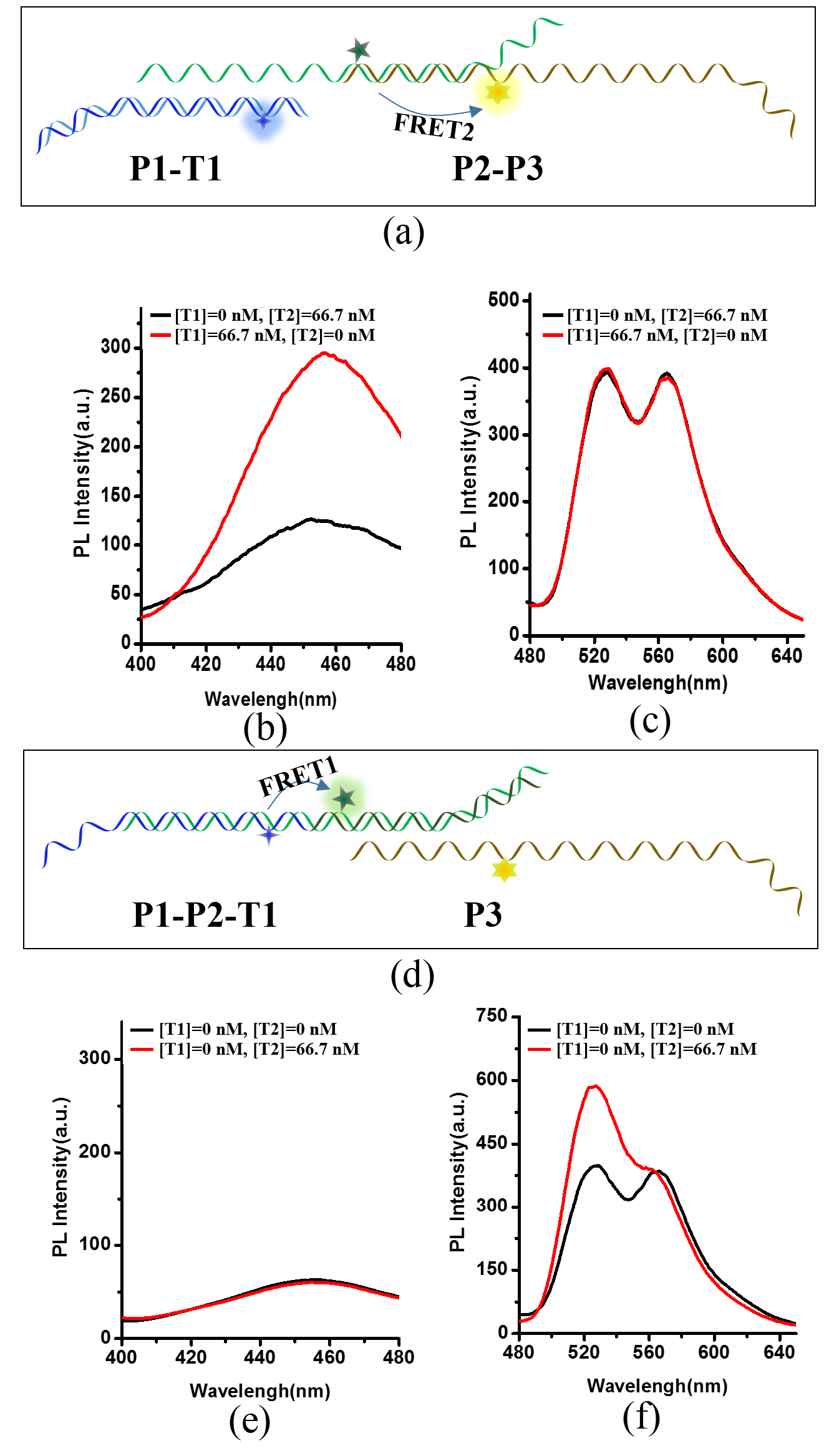


**Fig. S6** Schematic representation (a and d) and fluorescence spectra for two-step FRET detection system (P1-P2-P3) with different concentrations of target T1 (fig. b, c), T2 ( fig. e, f)). Excitation wavelengths: (b), (e) 364 nm; (c), (f) 457 nm.


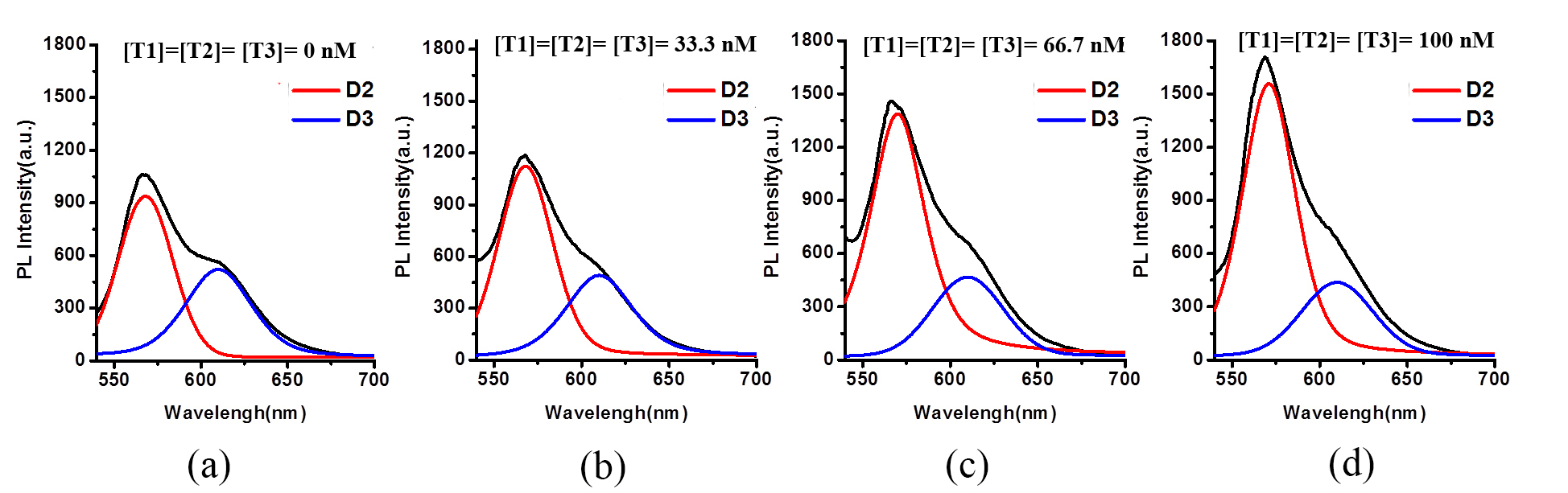


**Fig. S7** Fluorescence spectra of a four-DNA sequence assembled system (P1-P2-P3-P4) for the simultaneous detection procedures of various concentrations of targets (T1, T2 and T3). The experiment spectra (black curves) are fitted by a weighted summation of the constituent D3 and D4 emission spectra. The resulting weighted contributions of the individual dyes D3 (red curve, at 568 nm) and D4 (blue curve, at 610 nm) are shown. Excitation wavelengths: 520 nm.


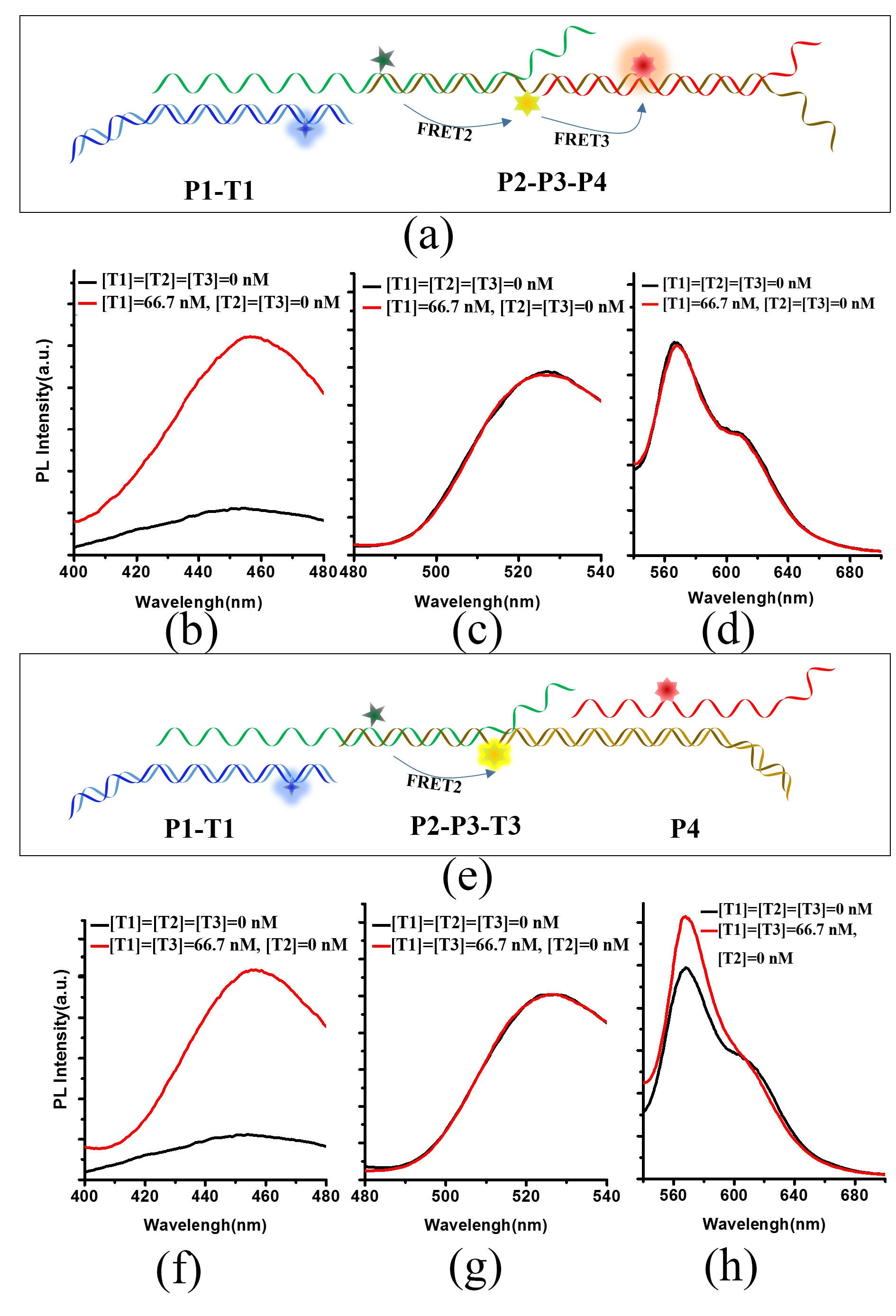


**Fig. S8** Schematic representation (a, e) and fluorescence spectra for three-step FRET detection platform (P1-P2-P3-P4) with different target T1 (fig. b, c, d), mixture of T1 and T3 (fig. f, g, h). Excitation wavelengths: (b), (f) 364 nm; (c), (g) 457 nm; (d), (h) 520nm.
